# Lumbar Interbody Cages: Design Characteristics, Biomaterials, Biomechanical Performance, Clinical Challenges, and Emerging Innovations

**DOI:** 10.7759/cureus.105284

**Published:** 2026-03-15

**Authors:** Adrian-Valentin Enache, Antonio-Daniel Corlatescu, Horia-Petre Costin, Alexandru Vladimir Ciurea

**Affiliations:** 1 Doctoral School, Carol Davila University of Medicine and Pharmacy, Bucharest, ROU; 2 Department of Neurosurgery, Carol Davila University of Medicine and Pharmacy, Bucharest, ROU

**Keywords:** 3d-printed titanium, expandable cages, lumbar fusion, lumbar interbody cages, osseointegration, peek, spinal implants, subsidence

## Abstract

Lumbar interbody cages have evolved significantly in recent decades through advances in biomaterials, geometric design, and surface modifications, all aimed at improving fusion and reducing complications. However, issues such as subsidence, migration, and pseudarthrosis continue to pose significant challenges in spinal practice.

This narrative review synthesizes current data on lumbar cage design, biomaterials used, biomechanical performance, clinical outcomes, and recent innovations. Current evidence suggests that 3D-printed porous titanium implants achieve higher fusion rates than polyetheretherketone and are associated with lower rates of subsidence and reintervention, although differences in patient-reported clinical outcomes remain small. Expandable cages offer radiographic advantages, especially in the restoration of disc height, but without a clear clinical superiority over static ones.

Overall, the choice of material, implant geometry, and surface properties significantly influence the performance of the cage. Innovations such as additive manufacturing, porous architectures, customized implants, and bioactive surfaces are promising, but long-term prospective studies are needed to more clearly define their role in individualized implant selection.

## Introduction and background

The primary goal of using interbody cages to perform lumbar interbody fusions is to help treat conditions that can include lumbar degenerative disc disease, spondylolisthesis, lumbar spine instability, and others, through the removal of the intervertebral disc and placing an implant (the interbody cage) between two vertebral bodies to increase disc space, create additional stability at the level of the fused vertebrae, and promote bony fusion of the two adjacent vertebrae. The development of the first interbody cages in the early 1980s has led to an advancement in technology related to interbody cages since their inception. These advancements include, but are not limited to, using new biomaterials, creating cages with better geometric designs, creating cages with increased porosity, and developing surface modification technologies to enhance fusion rates and decrease the likelihood of complications associated with interbody cage placement [1,2].

Although there is significant clinical history, as well as ongoing innovation in the design and application of interbody cages, they are still plagued with many issues, for both the surgeon and the patient, some of which may be multi-factorial in nature. Cage subsidence, cage migration, pseudarthrosis, and loss of sagittal correction are examples of complications that are commonly seen after lumbar interbody cage placements. The likelihood of experiencing one or more of these complications is significantly impacted by poor bone quality, specifically when it relates to osteoporosis and when intraoperative damage to either of the endplates of the vertebrae occurs, both of which negatively impact the structural integrity necessary for successful cage placement [3,4].

There are several factors that influence how a lumbar interbody cage will perform once implanted, including properties of the material of the cage, geometry of the cage, characteristics of the surface of the cage, surgical technique employed to place the cage, anatomy of the vertebrae, and quality of the bone into which the cage is being placed [5,6]. Therefore, the selection of an appropriate lumbar interbody cage should be viewed as part of a larger biomechanical and biological plan developed based on the specific needs of each patient and not simply as a decision between different product options.

Advancements in interbody cage design, including additive manufacturing, controlled porous structures, bioactive surface engineering, and patient-specific design, offer a greater variety of products than ever before; however, they also introduce new questions about clinical value, cost-effectiveness, and proper indications for use [7,8]. For this reason, we conducted a comprehensive narrative review of the current literature on lumbar interbody cages, focusing on biomaterials, cage design, biomechanical performance, clinical outcomes, and emerging technological innovations, with particular emphasis on recent comparative studies and meta-analyses.

This narrative review was based on a targeted literature search performed in PubMed, Scopus, and Google Scholar. The search focused primarily on studies published between 2020 and 2025, although seminal earlier articles were also included when necessary for historical context. Search terms included combinations of “lumbar interbody cage”, “lumbar fusion”, “PEEK”, “titanium”, “3D-printed cage”, “porous cage”, “expandable cage”, “static cage”, “subsidence”, “migration”, “fusion rate”, “osseointegration”, and “lordosis”. Priority was given to comparative clinical studies, meta-analyses, biomechanical studies, and recent reviews that were directly relevant to cage design, biomaterials, biomechanics, complications, clinical outcomes, and emerging innovations. This article is a narrative review and therefore does not follow a formal systematic review design.

## Review

Evolution of lumbar interbody cage design

Historically, static cages were used and inserted into the spine at a fixed height, determined prior to the procedure using preoperative disc space measurements and the surgical goals established by the surgeon. The concept of an expandable cage was developed to improve upon the limitations of the traditional static cage design. Expandable cages can be expanded post-operatively after insertion through a reduced surgical corridor, providing theoretical benefits of reducing endplate damage during the insertion process and increasing the restoration of the height of the intervertebral disc and indirectly providing neural decompression [9,10].

Several recent meta-analyses have compared the outcomes of static and expandable cages. Lee et al. evaluated thirteen studies with 1700 patients and concluded that expandable cages significantly increased anterior disc height and segmental lordosis when compared to static cages; however, they did not demonstrate any statistically significant difference in clinical outcomes, subsidence rates, or patient-reported outcome measures [1]. A 2021 meta-analysis conducted by Calvachi-Prieto et al. documented lower subsidence rates (3% vs. 27%) and greater restoration of disc height in the setting of minimally invasive procedures with expandable cages; however, these findings have not been consistently reproduced [4].

More recent data provide a more nuanced perspective regarding the performance of static versus expandable cages. Lai et al. retrospectively studied patients who underwent implantation of either static or expandable cages and reported significantly lower rates of cage migration (p = 0.01) and subsidence (p = 0.03) in the expandable cage group, along with improved leg pain relief and functional outcomes; however, a significantly higher rate of cage breakage was also observed (p = 0.04) [2]. In contrast, Kim et al. documented higher subsidence rates associated with expandable cages (14.7% vs. 3.9%, p = 0.047), although these devices provided superior restoration of disc height [3]. Similarly, a comprehensive meta-analysis by Daher et al. reported that static cages were associated with lower subsidence rates (OR = 0.50, p = 0.05) but higher reoperation rates (OR = 1.96, p = 0.01), whereas expandable cages demonstrated greater restoration of disc and foraminal height but inferior Oswestry Disability Index (ODI) scores at follow-up beyond six months [10]. However, it should be noted that many of these studies have relatively short follow-up periods, and despite differences in radiographic parameters, current evidence does not clearly demonstrate a consistent long-term clinical advantage of one cage type over the other.

The biomechanical rationale supporting expandable cages is based on their ability to reduce insertion forces on the vertebral endplates while increasing the implant-endplate contact area after expansion. Nevertheless, the expansion process itself may impose additional stress on the endplates and potentially compromise their integrity, particularly in patients with reduced bone quality such as osteoporosis, thereby limiting the theoretical advantages of expandable cage designs [11,12].

Current evidence indicates that expandable cages have radiographic advantages in terms of restoring disc height; however, they have few clinically proven advantages, with possible drawbacks including increased costs and the risk of device-specific failure modes.

Biomaterials used in lumbar interbody cages

Polyetheretherketone (PEEK) was introduced as an alternative to titanium in the 1990s, providing radiolucency for fusion evaluation, an elastic modulus closer to that of bone (thereby reducing stress shielding), and the absence of imaging artifacts [13,14]. However, PEEK's bioinert surface chemistry limits osseointegration; the potential for fibrous tissue formation at the bone-implant interface and delayed fusion are potential complications [15,16].

Titanium and titanium alloys have been employed in interbody fusion applications since their inception. Recent meta-analyses indicate that titanium cages exhibit superior fusion rates when compared to PEEK cages. Wang et al. examined 19 studies with 2,057 patients and demonstrated that titanium cages, including 3D-printed porous titanium, exhibited significantly greater fusion rates and fewer subsidence and reoperation events than PEEK cages [7]. Tan et al. indicated that titanium cages resulted in superior fusion in lumbar procedures (OR 2.12, 95% CI 1.05-4.28, p = 0.04); however, they also reported higher subsidence rates in non-infective cases [8].

Advancements in 3D printing technology have led to the development of porous titanium cages, which utilize titanium's osteoconductive properties combined with engineered porosity designed to facilitate bone ingrowth and reduce the elastic modulus mismatch. Several recent studies report fusion rates ranging from 86% to 93% at one year post-surgery with 3D-printed titanium cages compared to 67% to 82% with PEEK cages [17-19]. Yang et al. demonstrated 92.9% fusion at two years post-surgery with 3D-printed titanium cages compared to 82.3% with PEEK cages (p = 0.037), with no significant difference in subsidence rates [17]. A randomized controlled trial conducted in 2024 by Ham et al., employing non-window-type 3D-printed titanium cages (cages without a central void for bone graft), demonstrated 96.6% fusion rates; these findings suggest that the porous architecture of the 3D-printed titanium cages themselves may provide sufficient osseointegration without the need for supplemental bone graft [18].

Titanium-coated PEEK and hybrid designs aim to combine PEEK's biomechanical benefits with titanium's biological attributes. Hasegawa et al. published a multicenter randomized trial demonstrating that titanium-coated PEEK resulted in better bone fusion at six months post-surgery than uncoated PEEK (OR 2.27, 95% CI 1.09-4.74, p = 0.03) [11]. Although a 2025 meta-analysis conducted by Kincaid et al. demonstrated that titanium-coated PEEK resulted in superior fusion rates compared to uncoated PEEK in lumbar procedures, the differences were modest and procedure dependent [12].

Activated titanium cages employing surface modifications and activation techniques such as micro-etching, nano-topography, and microlattice structures have demonstrated promise. In a 2023 randomized controlled trial, Toop et al. demonstrated 84.0% versus 20.6% Bridwell Spinal Fusion (BSF) grade 3 fusion at six months post-surgery using activated titanium cages (nano-etched surface and microlattice structure) compared to PEEK cages (p < 0.001), respectively [9]. Additionally, the authors demonstrated significantly reduced subsidence rates (20.8% vs. 41.4%, p < 0.001) for the activated titanium cages [9]. The results of this study support the hypothesis that micro-scale surface features play a dominant role in early osseointegration.

Silicon nitride is a ceramic biomaterial with osteoconductive properties and antibacterial activity. While preclinical studies have demonstrated favorable bone apposition, long-term human case series have demonstrated exceptional osseointegration [20-22]. However, a 2017 randomized controlled trial comparing silicon nitride to PEEK in cervical fusions demonstrated equivalent clinical outcomes and fusion rates at 24 months post-surgery; these findings suggest that material advantages may not result in clinically relevant differences across all contexts [23].

Biodegradable cages produced from poly-L-lactide-co-D,L-lactide (PLDLLA) or polycaprolactone (PCL) composites have been studied as potential alternatives to traditional cages. Biodegradable cages may offer theoretical advantages by allowing them to resorb after fusion, thereby eliminating potential complications related to long-term implant presence. However, clinical experience has been less than encouraging. A 2009 randomized trial demonstrated significantly inferior fusion rates (p = 0.0302) and increased subsidence rates (p = 0.0414) with PLDLLA when compared to PEEK, with notable osteolysis present in some cases [24]. A 2010 study demonstrated 18.2% nonunion and 18.2% cage migration rates with biodegradable cages when compared to 0% with carbon fiber cages [25]. Although more recent 3D-printed polycaprolactone/β-tricalcium phosphate (PCL/β-TCP) composite cages have demonstrated superior results, with 95.2% fusion at 12 months in a small prospective series, longer-term follow-up remains limited [26].

Geometric and biomechanical considerations

Quantifying how loads are distributed among an instrumented lumbar spine is crucial to understanding instrumented spinal fusion cage function and likely failure mechanisms. Recent biomechanical studies employing instrumented cages and rods have quantitatively defined how forces are shared between cages, posterior instrumentation, and various anatomical structures [27,28].

Calek et al. reported that without posterior instrumentation, intervertebral cages absorbed >50% of axial load at every loading magnitude [28]. When posterior instrumentation was present, however, cages accounted for <25% of axial load at 100N, but this increased to 40-50% at 1000N. In addition, cages in bilateral posterior lumbar interbody fusion (PLIF) configurations typically absorbed more force than those in unilateral PLIF or transforaminal lumbar interbody fusion (TLIF) configurations. In addition, Khodaee et al. documented that anatomical structures consistently bore the greatest amount of axial load (mean 44.55% at 1000N), followed by cages (36.3%), and finally rods (14.44%) [27]. This highlights the need for maintaining structural anatomy intact during surgery.

The biomechanics of cage-endplate interactions is determined by contact area, the distribution of contact pressure, and the properties of the bony endplates. Yu et al. performed cadaveric experiments that illustrated how high volumetric bone mineral density (vBMD) and medial cage positioning resulted in higher contact pressures, whereas low vBMD and transverse cage orientation resulted in larger contact areas [29].

Finite element models of spinal cages have consistently shown that larger cage footprints reduce the maximum endplate stresses experienced by cages, whereas the stiffness of the cage material can influence stress shielding and the manner in which loads are transferred from one structure to another [30,31]. Umale et al. documented that bilateral posterior instrumentation (TLIF, PLIF, circumferential lumbar fusion 360° fusion [CLIF/360]) most effectively restricted the range of motion of the instrumented level, but the degree of restriction was inversely related to the endplate stresses experienced by the cages and directly related to the cross-sectional area of the cages, thereby representing a mechanism of subsidence [32].

Loads at the cage-endplate interfaces are influenced by the size and stiffness of the cages, the geometry of the bony endplates, and the presence of adjacent instrumentation. Recent biomechanical investigations have employed instrumented cages and rods to quantify load distributions in instrumented lumbar fusions, providing new insights into cage performance and potential failure mechanisms [27,28].

Calek et al. investigated the role of posterior instrumentation on cage-endplate load distribution using finite element modeling. The results indicated that when no posterior instrumentation was present, intervertebral cages absorbed >50% of axial load at every loading magnitude [28]. By contrast, when posterior instrumentation was included, cages only absorbed <25% of axial load at 100N; however, this proportion of axial load absorbed by the cages increased to 40-50% at 1000N. Additionally, they found that cages in bilateral PLIF configurations tended to absorb more force than those in unilateral PLIF or TLIF configurations [28].

Khodaee et al. further elucidated the role of instrumentation in load distribution by documenting that anatomical structures consistently bore the greatest proportion of axial load (mean 44.55% at 1000N), followed by cages (36.3%), and then rods (14.44%) [27]. These findings highlight the need to maintain anatomical structures intact during surgery.

Yu et al. demonstrated how the mechanical properties of the bony endplates interact with cage-endplate contact mechanics using cadaveric experiments. The results showed that cages positioned medially and supported by bony endplates with high vBMD resulted in higher contact pressures, whereas cages oriented in a transverse plane and supported by bony endplates with low vBMD resulted in larger contact areas [29]. Therefore, it is reasonable to expect that cage selection and positioning should be based on the individual's bone quality to optimize load distribution between the cage and surrounding bony structures.

Finite element analyses have consistently demonstrated that larger cage footprints result in lower peak endplate stresses [30], whereas cage material stiffness has been shown to influence stress shielding and the pattern of load transfer among the structures involved in the fusion [32]. Umale et al. documented that bilateral posterior instrumentation (TLIF, PLIF, CLIF/360) most effectively restricted the range of motion of the instrumented level; however, the degree of restriction was inversely related to the endplate stresses experienced by the cages and directly related to the cross-sectional area of the cages, thus representing a mechanism of subsidence [32].

The primary goal of spinal surgery involves restoring disc height and segmental lordosis, providing indirect neural decompression, and achieving proper sagittal balance and clinical results. There are multiple variables that will affect how much of each are restored, such as cage height, lordotic angle, cage position, the surgical method used to place the cage, and the use of posterior column release [33-36].

In an experimental study using cadavers, Robertson et al. reported that a posterior column osteotomy (PCO) was associated with the greatest amount of lordosis achieved in the TLIF procedure. They found that mean lordosis increases of 2.2° were achieved with each PCO performed during the TLIF procedure [36]. It was noted that when PLIF cages were placed bilaterally and were shorter than those used in TLIF procedures, lordosis restoration was significantly greater in cases where the cages were placed unilaterally and were longer [36]. Therefore, it can be concluded that cage length as well as whether the cages are placed bilaterally will contribute to the rotational mechanics.

There is a non-linear relationship between cage lordotic angle and the amount of segmental lordosis achieved. While cages with lordotic angles of 15° are generally more effective than cages with lower lordotic angles in achieving lordosis restoration, the amount of lordosis restoration is influenced by cage positioning, preoperative disc height, and the specific surgical technique used [33,34]. Placement of the cage anteriorly can maximize the lordotic effect due to the increase in the moment arm; however, this placement method is often limited by both the surgical approach used and anatomically [35].

While theoretical advantages of expandable cages include maximizing disc height restoration via maximal expansion post-cage insertion, meta-analysis has confirmed that superior anterior disc height restoration occurs with expandable cages compared to static cages (standardized mean difference = 0.478, p = 0.0162). However, there were no significant differences in posterior disc height and lumbar lordosis restoration with the use of expandable versus static cages [10]. The clinical relevance of these radiographic findings remains unclear, as patient-reported outcomes did not reveal any clinically relevant differences between the two cage types.

For successful interbody fusion to occur, osseointegration at the cage-endplate interface must first take place, followed by the formation of bridging bone across the interbody space. The physical properties of the material from which the cage is made, the surface characteristics of the cage, and the architectural design of the cage all play a significant role in influencing the above-mentioned biological processes [37-40].

Due to its bio-inert surface, PEEK does not allow for protein adsorption and subsequent osteoblast attachment and therefore leads to fibrous encapsulation rather than direct bone apposition [15,38]. In contrast, the oxide layer formed on the surface of titanium allows for favorable surface chemistry for protein adsorption and cell attachment, leading to osseointegration [37,40]. Osteoblast adhesion, proliferation, and differentiation have also been shown to be enhanced with various surface modification techniques such as micro-arc oxidation, hydrothermal treatment, and nano-etching [9,41].

The geometry of the cage is a primary factor influencing both the biomechanical performance and the restoration of sagittal alignment in the case of lumbar interbody fusion. The size of the cage footprint and the extent of endplate coverage will determine how loads are distributed and therefore affect the risk of subsidence; wider cages (≥22 mm) have been found to be superior at providing support for the endplates and reducing the rate of subsidence [42]. The results from biomechanical studies demonstrate that cages covering the apophyseal ring will result in a reduction in endplate stresses and improve the uniformity of loading across the entire vertebral body [43,44]. The lordotic angle built into the cage will contribute to the restoration of the lordosis at the segmental level; however, the overall sagittal balance is determined by both the design of the implant and the position of the implant within the disc space [45]. The heights of the cages and the anterior-posterior positions also greatly impact sagittal balance, with anterior placements generally resulting in greater corrections to the segmental lordosis [46,47]. As these factors are highly relevant when dealing with patients with poor bone quality, proper cage size and placement can help reduce endplate stress and subsequently reduce the risk of subsidence [48,49]. Overall, the geometry of the implants plays a significant role in determining the radiographic outcomes related to disc height restoration and segmental alignment, which have direct correlations to post-operative clinical outcomes [50].

Complications and failure mechanisms

Bone ingrowth into porous cages occurs through the pores themselves, with optimal pore diameters of 300-600 μm for vascular invasion and osteogenesis. Furthermore, 3D-printed titanium cages with controlled porosity (usually 60-70%) demonstrate elastic modulus values closer to bone while maintaining structural integrity, thus reducing stress shielding and enhancing osseointegration. The presence of trabecular bone remodeling signs seen on CT scans in cages with successful fusions indicates active bone remodeling within the porous structure [17-19,37,38].

Various bioactive coatings, including hydroxyapatite, calcium phosphate, and growth factors, have been studied to enhance osseointegration. In a study of sheep models, Sun et al. demonstrated that a combination of micro-arc oxidation and hydrothermal treatment produced a bioactive calcium phosphate coating with micro/nano morphology that enhanced osseointegration and reduced the failure rate of fusions. However, the application of bioactive coatings in humans has been limited, and most of the current evidence comes from preclinical studies [41].

The traditional concept of bone graft being necessary for fusion in cages is now being challenged based on recent evidence regarding the use of 3D-printed titanium cages. Ham et al. conducted a study and reported no difference in fusion rates between window-type cages (that had a bone graft void) and non-window-type cages (96.6% vs. 93.8%). Additionally, they reported that the surface osseointegration scores of the non-window cages were higher. These findings challenge the traditional assumption that bone graft is always required for successful fusion and suggest that optimized cage surface properties may allow adequate osseointegration even without supplemental bone graft [18].

Subsidence, migration, and loss of correction are among the most common cage-related complications following spinal arthrodesis. The literature reports a wide range of reported incidence for cage subsidence (ranging from 3-58%) based on the definition used to define cage subsidence, the surgical technique employed, and the specific patient population [13-15,42].

Extensive research has investigated the risk factors for cage subsidence. These include patient-related factors such as low bone mineral density (BMD), elderly status, female sex, elevated BMI, and comorbid conditions [13-15]. Yao et al. identified BMD, disc height, and cage placement as the three major negative risk factors for cage settling in minimally invasive TLIF [13]. Ran et al. showed that Hounsfield units (HU) measured using pre-operative CT scans can predict the risk of cage subsidence, with optimal threshold values being 115 HU for upper instrumented vertebrae and 125 HU for lower instrumented vertebrae [15].

Surgical and implant-related factors contributing to cage subsidence include endplate damage, cage size, cage material, and cage positioning [13,14,42]. Wu et al. conducted a systematic review of lateral lumbar interbody fusions and identified multiple factors that contribute to increased cage subsidence, including poor bone quality, multi-level procedures, narrow cage widths (<22 mm), and tall cage heights (>11 mm) [14]. Subramanian et al. documented that cage width is predictive of cage subsidence in expandable cages; specifically, a cage width of <24 mm resulted in a 14.9% overall subsidence rate [49].

The relationship between cage material and cage subsidence is currently under investigation. While some studies have reported increased cage settling with titanium due to greater elastic modulus mismatch between titanium and the bony endplates, other studies have reported reduced cage settling with 3D-printed titanium cages due to superior osseointegration [8,17,19]. A 2025 meta-analysis of cage materials found that 3D-printed titanium cages demonstrated significantly lower cage subsidence rates at one year post-operatively when compared to PEEK cages (p<0.05); therefore, it appears that osseointegration may be the dominant factor in cage stability and not the elastic modulus difference between the cage material and the endplates [19].

While cage migration, defined as posterior displacement of the cage into the spinal canal, is less common than cage subsidence, it is generally considered to be a more severe complication of cage placement. Cage migration has been reported to occur in approximately 1.6-2.4% of patients in recent series [51-54]. Factors that increase the risk of cage migration include osteoporosis, endplate damage, a "pear-shaped" disc morphology, posterior cage placement, and the use of single unilateral cages [52,53]. Wang et al. identified several factors that increase the risk of cage migration, including screw loosening (OR 12.98), endplate damage (OR 10.14), and pear-shaped discs (OR 4.03); in addition, they noted that PEEK cages may reduce the risk of cage migration (OR 0.51) [51].

There is an ongoing debate regarding the clinical implications of cage subsidence and migration. Rickert et al. evaluated 57 TLIF patients and determined that cage migration occurred in 85.1% and cage subsidence in 58.2%; however, neither cage migration nor cage subsidence impacted either fusion rates or clinical outcomes [16]. Therefore, minor degrees of cage settling may be indicative of normal biomechanical adaptation and not necessarily a sign of cage failure. However, significant cage subsidence (>3-5 mm) or symptomatic cage migration clearly correlates with decreased clinical outcomes, loss of indirect decompression, and increased reoperation rates [2,51].

Clinical evidence and comparative outcomes

Recent meta-analyses have assessed the question of whether interbody cages are more effective than structural bone graft alone. Johnson et al. in their 2024 meta-analysis evaluated 20 studies and 1,508 patients and found that when cages were used instead of structural bone graft, there were statistically significant increased fusion rates (96.3% vs. 90.8%, p = 0.03), increased disc height preservation (4.0 vs. 3.4 mm, p = 0.001), and decreased back pain (visual analog scale [VAS] score 5.4 vs. 4.7, p = 0.03) [55]. These data provide further evidence supporting the clinical utility of using interbody cages in lumbar arthrodesis.

When comparing different cage materials, recent data clearly demonstrate that titanium, particularly 3D-printed porous titanium, is superior to PEEK for achieving fusion. Multiple meta-analyses have demonstrated that titanium cages result in a 5-10% increase in fusion rates as compared to PEEK cages, with this difference being most notable for 3D-printed titanium cages. However, while these radiographic benefits appear to translate to improved clinical outcomes (measured by ODI and VAS), the differences seen clinically are relatively small [8,10].

There are two major limitations of contemporary data assessing cage performance: 1) the majority of the evidence is comprised of retrospective studies and 2) most studies evaluate outcomes after one to two years, which is too early to determine if long-term fusion quality will be maintained, if adjacent segments will undergo degenerative changes, or if late complications will occur. Furthermore, the variability in how fusion and subsidence are defined, as well as how outcomes are measured and reported, makes it difficult to synthesize the results of multiple studies and interpret them in a meaningful clinical manner [56].

The static versus expandable cage controversy illustrates the challenges in demonstrating that theoretically derived biomechanical advantages translate into clinical benefits. There are several intuitively appealing features of expandable cages: they allow for a smaller insertional diameter, thereby decreasing the potential for nerve root retraction, can maximize disc height restoration as they expand in place, and may reduce endplate damage during the insertion process [9,10].

Despite these theoretical advantages, multiple meta-analyses demonstrate a disconnection between the radiographic and clinical results obtained using expandable cages. While all of the meta-analyses demonstrate that expandable cages have superior disc height and foraminal height restoration compared to static cages, none of the meta-analyses demonstrate any clinically relevant differences in patient-reported outcome measures. In fact, the 2023 meta-analysis by Lee et al. concluded that expandable cages do not demonstrate a clinically significant improvement in patient outcomes when compared to static cages despite the radiographic advantages of expandable cages [1]. A 2025 meta-analysis by Daher et al. also demonstrated that at ≥6 months post-operatively, static cages had better ODI scores (mean difference 3.77, p = 0.03) than expandable cages, raising questions regarding whether the additional expense of expandable cages provides an advantage [10].

While complication profiles differ between cage designs, the potential for specific complications differs between the two designs. Both expandable and static cages present specific advantages and limitations that should be interpreted within the broader biomechanical and clinical context. Expandable cages may reduce insertion-related endplate stress and, in some cases, demonstrate lower migration rates, although higher subsidence rates and device-specific complications such as cage breakage or mechanical failure of the expansion mechanism have also been reported [2,3]. Static cages, while mechanically simpler and associated with fewer implant-specific failure modes, have been linked in some studies to higher reoperation rates [6]. These findings suggest that cage selection should not be framed as a simple comparison between two competing designs. Instead, the choice of implant should be individualized based on factors such as patient anatomy, vertebral endplate integrity, bone quality, and the desired sagittal alignment and biomechanical objectives of the procedure.

Emerging innovations and future directions

In addition to enhancing geometrical and mechanical properties of cages via 3D printing, the ability to control the amount of porosity as well as generate an internal structure, which would be difficult to produce via traditional manufacturing, has enhanced cage design. A major advantage of the 3D-printed cages made of titanium with a high degree of porosity (usually 60-70%) is that they have an elastic modulus of approximately 1-5 GPa, which is relatively close to the modulus of cortical bone (10-20 GPa), whereas the elastic modulus of pure titanium is much larger (110 GPa). As such, the degree of stress shielding that occurs when a cage is implanted into the spine is decreased; however, the mechanical strength of the cage remains intact [17-19,37,38,57-60].

Recent clinical trials have demonstrated significant improvement in fusion rates with the use of 3D-printed titanium cages. Several recent studies have documented fusion rates ranging from 86% to 96% at one year post-operatively with the use of these cages and that this was substantially greater than that obtained with the use of PEEK cages (67-82%). Yang et al. noted that the fusion rate at two years post-operatively was 92.9% for patients who had received 3D-printed titanium cages versus 82.3% for those who had received PEEK cages (p = 0.037) [17]. Jacob et al. reported that the fusion rate at three years post-operatively was 93.3% for patients who had received 3D-printed titanium cages versus 73.2% for those who had received titanium-coated PEEK cages (p < 0.0001); furthermore, there were statistically significant differences in the degree of subsidence observed with these two groups of patients (6.0% vs. 25.0%, p < 0.0001) [58].

Bone ingrowth into the cage is facilitated by the porous structure generated during the printing process, and the pore size generated in the cage is critical for vascular invasion and osteogenesis. Optimal pore size for this purpose ranges from 300 to 600 microns [37]. Turlip et al. documented a fusion rate of 90.3% for lateral interbody fusion (LLIF) and a fusion rate of 81.3% for anterior lumbar interbody fusion (ALIF) with the use of a new type of 3D-printed titanium cage system; furthermore, they noted a statistically significant improvement in both ODI and Patient-Reported Outcome Measures Information System (PROMIS) scores with the use of this cage system [57].

One of the most paradigm-changing findings of the past few years regarding the use of 3D-printed titanium cages has been reported by Ham et al., who showed that non-window-type 3D-printed titanium cages can achieve a fusion rate of 96.6%, which is equivalent to that of window-type cages (93.8%) [18]. This suggests that if the porous structure of a cage can be optimized, then the need for bone graft may be eliminated, which could simplify surgery and reduce the morbidity associated with either harvesting or procuring a bone graft.

Using computational topology optimization in combination with additive manufacturing technology allows for the development of patient-specific cages that can be designed to fit anatomically and mechanically to each specific patient's anatomy and bone quality [58-60]. Smit et al. developed a full-scale topology optimization formula that accounted for the structural response of adjacent bone to develop cages that reduced the risk of subsidence of the cage by 89% for titanium and 94% for PEEK relative to that of off-the-shelf cages [59,60].

With patient-specific cage design, it is possible to optimize multiple objectives simultaneously, including increasing the footprint of the cage within anatomical constraints, adjusting the elastic modulus of the cage to match the local bone quality, optimizing the lordotic angle of the cage for sagittal balance goals, and decreasing stress concentrations at the cage-endplate interface [31,57,60]. Weng et al. proposed creating customized cage porosity based on the volumetric BMD of the endplates measured prior to surgery via preoperative CT scans to create a biomechanical match between the cage and the bone [47].

Although theoretically advantageous, the practical application of patient-specific cages will likely be limited due to several factors, including regulatory pathways for custom devices, manufacturing lead times incompatible with emergent surgical situations, costs, and the lack of clinical outcomes that demonstrate superiority to optimized off-the-shelf cage designs. Currently, patient-specific cages are being applied to complex deformity cases and revision surgeries where standard cages cannot provide adequate support [59,60].

Surface modification strategies aim to improve osseointegration while maintaining the favorable properties of the base material. Among the main approaches are the modification of topography at the micro- and nano-scale, the application of bioactive coatings, the incorporation of growth factors, and the development of surface treatments with an antimicrobial role [37-41].

Surface changes at the micro- and nanometer scale influence protein adsorption, cell adhesion, and osteoblastic differentiation. Toop et al. demonstrated that nano-etched titanium with microlattice structure achieved a BSF grade 3 fusion rate of 84.0% at six months compared to 20.6% for PEEK (p<0.001), supporting the hypothesis that microscopic surface properties play a key role in early osseointegration [9]. Bioactive coatings, such as hydroxyapatite, calcium phosphate, and bioactive glass, promote bone apposition. Sun et al. showed that micro-arc oxidation combined with hydrothermal treatment allowed the achievement of calcium phosphate-based bioactive coatings, which accelerated osseointegration and reduced fusion failure in an experimental model in sheep [41]. Titanium-coated PEEK is another form of bioactive modification already clinically available, and evidence from randomized trials has demonstrated an improvement in fusion at six months compared to uncoated PEEK [11].

Antimicrobial changes to the surface try to reduce the risk of implant-associated infections, one of the most severe complications of spinal surgery. Silver nanoparticles, copper ions, and antimicrobial peptides have been investigated for this purpose, although their transposition into clinical practice remains limited. Laubach et al. proposed surface concepts that combine osteoconductive and antimicrobial properties, but human data are lacking for now [38]. In parallel, the incorporation of growth factors, including bone morphogenetic protein-2 (BMP-2), has been intensively studied but remains controversial due to potential complications such as ectopic bone formation, radiculitis, and high costs [57]. Controlled-release systems and the use of lower doses could reduce these risks while maintaining the osteoinductive benefits, but optimal formulations have not yet been defined.

The future of lumbar interbody cages could involve a paradigm shift, from permanent implants to bioresorbable structures capable of facilitating fusion and then resorption, thus eliminating the complications associated with long-term implants [38]. Although the first biodegradable cages, such as those in PLDLLA, had disappointing clinical results, recent advances in materials science and additive manufacturing technologies have brought this direction back into focus [24,25].

In this context, medical polycaprolactone (mPCL) composites with the addition of ceramic phases exhibit favorable degradation kinetics, mechanical properties close to those of cortical bone, and excellent biocompatibility. Liu et al. reported a fusion rate of 95.2% at 12 months for PCL/β-TCP 3D-printed cages, with partial resorption observed in all cases [26]. However, long-term data, beyond two years, remain limited, and concerns about osteolysis and late mechanical failure persist. Another biodegradable alternative is magnesium-based alloys, which have an elastic modulus closer to that of bone and osteogenic properties. However, controlling the rate of degradation and managing the release of hydrogen during corrosion remain challenges that require further research [39].

**Figure 1:**
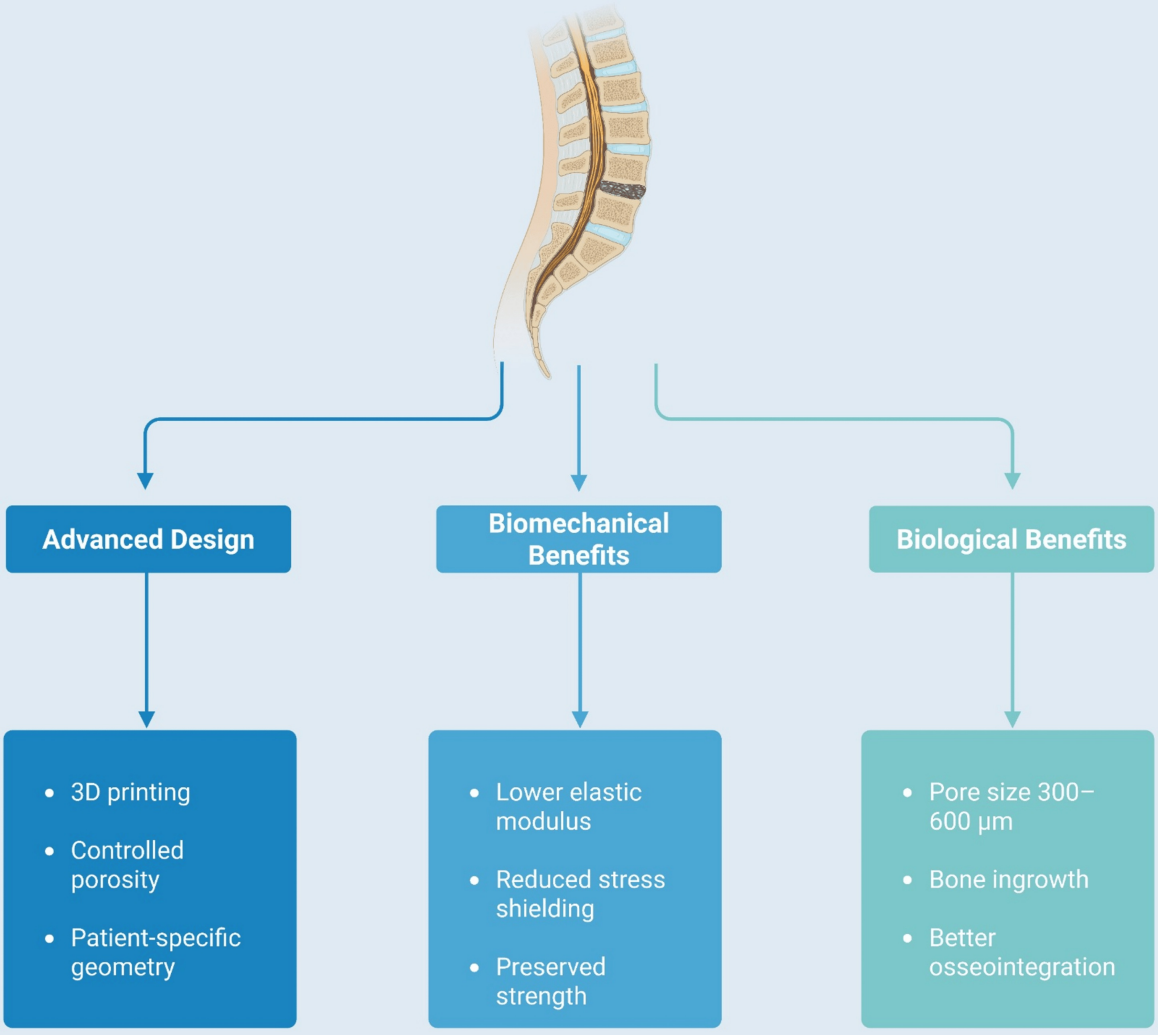
Conceptual overview of the advantages of next-generation lumbar interbody cage design Advances in cage engineering, particularly through 3D printing, controlled porosity, and patient-specific geometric customization, contribute to both biomechanical and biological improvements in lumbar interbody fusion. From a biomechanical perspective, these design strategies enable reduction of the effective elastic modulus, mitigation of stress shielding, and preservation of mechanical strength. From a biological perspective, optimized porous architecture, including pore sizes in the range of 300–600 μm, promotes bone ingrowth and improved osseointegration. Together, these features illustrate how modern cage design aims to better match implant behavior to the mechanical and biological requirements of the spinal fusion environment. This is an original figure created by the authors using BioRender.com.

In parallel, the integration of smart technologies, including sensors for real-time fusion monitoring, controlled drug or growth factor delivery systems, and mechanically adaptive materials capable of altering their stiffness during healing, outlines a futuristic perspective. Although these directions seem technologically feasible, their real clinical need, regulatory pathways, and cost-effectiveness still remain uncertain [39] (Figure 1).

## Conclusions

Lumbar interbody cages have evolved considerably from simple structural devices to increasingly sophisticated implants, developed through advances in biomaterials, geometric design, and surface engineering. Current evidence suggests that these innovations have improved fusion performance and expanded surgical options, but without demonstrating the existence of a superior universal model. Material properties, implant geometry, and surface characteristics directly influence cage behavior, especially with regard to osseointegration, load distribution, disc height restoration, and segmental alignment correction. However, the radiographic advantages do not always translate into a clear clinical benefit, which remains an important limitation in the interpretation of the current literature.

Among the most relevant observations is the fact that porous titanium cages obtained by 3D printing seem to provide better fusion in the early phase compared to conventional PEEK implants, while expandable cages can provide a more efficient radiographic restoration in certain selected situations. However, these advantages are often accompanied by minimal differences in patient-reported clinical outcomes and distinct complication profiles, which shows that new technologies cannot automatically be considered superior. Subsidence and migration remain major challenges, being influenced by the interaction between factors related to the implant, the surgical technique, and the characteristics of the patient, such as bone quality, preparation of the vertebral plateaus, cage dimensions, and its positioning. In parallel, surface modifications and porous architectures emerged as promising strategies to improve biological integration and accelerate fusion.

Overall, the available data suggest that the choice of the optimal lumbar interbody cage depends on the clinical context and cannot be reduced to a universally valid model. Implant selection should be guided by the interaction between the patient's anatomy, bone quality, underlying pathology, surgical approach, and biomechanical goals of the intervention. Future progress in this area is likely to depend less on the identification of a single ideal cage and more on the development of evidence-based, individualized selection strategies supported by prospective comparative studies, long-term follow-up, and cost-effectiveness analyses.
